# [Expression of Concern] Small interfering-high mobility group A2 attenuates epithelial-mesenchymal transition in thymic cancer cells via the Wnt/β-catenin pathway

**DOI:** 10.3892/ol.2025.15222

**Published:** 2025-08-11

**Authors:** Sheng Tan, Jili Chen

**Affiliations:** Department of Cardiovascular Surgery, Affiliated Hospital of Xuzhou Medical University, Xuzhou, Jiangsu 221000, P.R. China; Department of Ophthalmology, Affiliated Hospital of Xuzhou Medical University, Xuzhou, Jiangsu 221000, P.R. China

Oncol Lett 22: 586, 2021; DOI: 10.3892/ol.2021.12847

Following the publication of this paper, it was drawn to the Editor's attention by a concerned reader that, for the cell migration and invasion assay data shown in Fig. 4C, the ‘si-HMGA2 / Migration’ panel appeared to contain an overlapping section of data with the ‘Control / Invasion’ panel, suggesting that these data had been derived from the same original source, even though the results from differently performed experiments were intended to have been portrayed.

The authors were contacted by the Editorial Office to offer an explanation for this apparent anomaly in the presentation of the data in this paper; however, up to this time, no response from them has been forthcoming. Owing to the fact that the Editorial Office has been made aware of potential issues surrounding the scientific integrity of this paper, we are issuing an Expression of Concern to notify readers of this potential problem while the Editorial Office continues to investigate this matter further.

